# Knowledge and Awareness of Amblyopia Among the Adult Population of the Al-Baha Region, Saudi Arabia

**DOI:** 10.7759/cureus.73374

**Published:** 2024-11-10

**Authors:** Mahadi Bashir, Sarah S Taishan, Bushra M Alghamdi, Rawan S Alzahrani, Jana R Khan, Ibrahim M Alamri, Hassan S Alomari, Lujain A Alghamdi

**Affiliations:** 1 Ophthalmology, General Surgery, Faculty of Medicine, Al Baha University, Al Baha, SAU; 2 Faculty of Medicine/Surgery, Al-Baha University, Al-Baha, SAU

**Keywords:** amblyopia, awareness, eye diseases, prevalence, saudi arabia

## Abstract

Background: Amblyopia results from abnormal visual development, leading to reduced vision. Early recognition of the disease can facilitate early treatment and increase the chances of recovering visual acuity. Despite its prevalence and treatable nature, there is a concerning lack of awareness about amblyopia among the general population in Saudi Arabia. This study aimed to assess amblyopia awareness among adults in the Al-Baha region of Saudi Arabia.

Methodology: A cross-sectional study was conducted among the general population of the Al-Baha region in Saudi Arabia from August 10, 2024, to October 1, 2024. Exclusion criteria included individuals who were not residents of the specified area and those under 18 years of age. Data were collected using a validated electronic questionnaire that covered sociodemographic information and characteristics of amblyopia.

Results: A total of 400 individuals participated, exceeding the target sample size of 384. Participants' ages ranged from 18 to over 50 years, with the majority being 18-29 years (245 participants, 61.3%). Females comprised the largest group, accounting for 246 (61.5%) of the participants. The majority of participants demonstrated a low level of knowledge regarding the concept of amblyopia. There was no significant association between various sociodemographic characteristics and the level of knowledge, except for participants with a family history of eye diseases. Interestingly, participants without a family history of eye diseases exhibited a higher level of knowledge than those with a family history (P = 0.03).

Conclusion: The majority of participants exhibited a low level of knowledge about amblyopia, highlighting the need for increased public awareness campaigns about amblyopia in the Al-Baha region of Saudi Arabia.

## Introduction

Amblyopia, commonly known as "lazy eye," is a cortical visual impairment affecting 3%-5% of the population [1,2]. It results from abnormal visual development in childhood, leading to reduced vision, typically in one eye. Amblyopia can be caused by abnormal binocular interaction, uncorrected refractive errors, or media opacities, while treatment involves improving blurred vision and encouraging use of the amblyopic eye through occlusion or atropine penalization of the better-seeing eye [3]. The condition is largely reversible during the critical period of visual development, typically the first seven to eight years of life [1]. Early detection through vision screening is crucial for successful treatment and the prevention of permanent vision loss [3].

The pooled estimate of amblyopia prevalence among children in Saudi Arabia is 2.3% [4]. This prevalence varies across regions and age groups, with older children experiencing higher rates. Notably, unilateral amblyopia is more prevalent than bilateral amblyopia [5].

Recent studies in Saudi Arabia have revealed a concerning lack of awareness about amblyopia among adults. In Arar, only 39% of participants correctly understood amblyopia treatment options [6]. Similarly, a nationwide study found that 70% of parents had no prior knowledge of amblyopia [7]. In Riyadh, only 51.3% of parents were aware of lazy eye, with limited understanding of treatment timelines [8]. This lack of awareness extends to healthcare settings, with a study in Jeddah reporting only 49.7% amblyopia awareness among pediatric and ophthalmology clinic attendees [9]. Misconceptions were common, such as the belief that amblyopia could be detected with the naked eye [6]. A study conducted in the Al-Baha region found that more than 50% of parents lacked awareness regarding the causes, risk factors, diagnosis, and management of amblyopia [10].

Unfortunately, existing literature in Saudi Arabia has demonstrated a notable lack of awareness about amblyopia among the general population across all regions. To the best of our knowledge, no research has been undertaken to assess the level of awareness regarding amblyopia among the adult population in Al-Baha. The primary aim of this study is to assess the level of amblyopia awareness among adult individuals in Al-Baha, Saudi Arabia.

## Materials and methods

Study design

This cross-sectional survey-based study was conducted from August 10, 2024, to October 1, 2024, among the general population of the Al-Baha region in Saudi Arabia to assess public knowledge and awareness of amblyopia. The study included all residents of the Al-Baha region aged 18 to 80 years who agreed to participate. Individuals who did not reside in the Al-Baha region, who were under 18 or above 80 years old, or who did not consent to participate were excluded.

Ethical approval and informed consent

The study was approved by the Institutional Research Board of Al-Baha University (approval number: REC/SUR/BU-FM/2024/61) on May 29, 2024. Prior to participation, all participants were informed about the study's aims and assured of data confidentiality. Consent was obtained from all participants.

Sample size calculation

The sample size was calculated using the Raosoft sample size calculator (Raosoft, Inc., Seattle, Washington), which uses the following formula:

$n = \frac{z^{2} * p * (1 &minus; p) / e^{2}}{1 + (z^{2} * p * (1 &minus; p) / (e^{2} * N))}$

with a precision level of ±5% and a confidence level of 95%. The estimated population of the Al-Baha region was 487,108, and the calculated sample size was 384. The study enlisted 400 participants.

Data collection and management

Data were collected from study participants using an online, anonymous, self-administered, reliable, prevalidated, and modified questionnaire derived from a previous study entitled “Awareness of Amblyopia Among the Adult Population in Arar City of Saudi Arabia” conducted by Parrey et al. [6]. The questionnaire was translated into Arabic and disseminated among the general population of the Al-Baha region using social platforms, such as WhatsApp and Telegram. All participants were provided detailed information about the study’s aims and data confidentiality.

Statistical analysis

Data were coded and entered into IBM SPSS Statistics for Windows, Version 26 (Released 2019; IBM Corp., Armonk, New York). The questionnaire consisted of five sections: Section 1 covered demographics and personal information; Section 2 evaluated participants’ understanding and awareness of the lazy eye concept; Section 3 focused on common causes; Section 4 measured knowledge of treatment options; and Section 5 addressed amblyopia complications. Frequency and percentage were used to describe the results for all questions. For the knowledge and causes sections, participants were awarded one point for each correct answer, resulting in a score range from 0 to 5, with a score of 3 or higher considered adequate. For the complications and treatment sections, the score ranged from 0 to 6, with a score of 4 or higher considered adequate. A chi-square test was used to determine the relationship between knowledge and demographic factors. All statements with a P-value lower than 0.05 were considered significant.

## Results

The study included 400 participants, with a higher proportion of females (246, 61.5%) compared to males (154, 38.5%). The majority were aged 18-29 years (245, 61.3%), followed by those aged 40-49 years (62, 15.5%). Residency distribution showed that 145 (36.3%) were from Al-Baha. Most were single (233, 58.3%). Educational levels varied, with the majority holding a college bachelor’s degree (266, 66.5%), followed by high school graduates (86, 21.5%). Monthly income was predominantly less than 5000 Saudi Riyal (241, 60.2%), with 76 (19%) earning between 5000 and 10,000 Saudi Riyal. A larger portion (276, 69%) did not have children under 12 years old, and only 22 (5.5%) had a child with lazy eyes. Additionally, 127 (31.8%) reported a family history of eye diseases (Table 1).

**Table 1:** Sociodemographic characteristics of the population of the Al-Baha region

Demographic Variables	Response	N (%)
Gender	Male	154 (38.5)
	Female	246 (61.5)
Age group (years)	19–29	245 (61.3)
	30–39	60 (15)
	40–49	62 (15.5)
	Above 50	33 (8.3)
Residency	Al-Baha	145 (36.3)
	Beljurashi	51 (12.8)
	Almakhwat	65 (16.3)
	Al-Aqiq	65 (16.3)
	Others	74 (18.5)
Marital status	Single	233 (58.3)
	Married	161 (40.2)
	Divorced	6 (1.5)
Education level	Primary	15 (3.7)
	High school	86 (21.5)
	College bachelor	266 (66.5)
	Master or PhD	32 (8)
	Illiterate	1 (0.3)
Monthly income	Less than 5000	241 (60.2)
	5000–1000	76 (19)
	10,000–15,000	48 (12)
	More than 15,000	35 (8.8)
Do you have a child under 12 years old?	Yes	124 (31)
	No	276 (69)
Do you have a child with lazy eyes?	Yes	22 (5.5)
	No	378 (94.5)
Do you have a family history of eye diseases?	Yes	127 (31.8)
	No	273 (68.2)
Disease	Hypermetropia or myopia	63
	Astigmatism	19
	Ptosis	8
	Cataract	12
	Glaucoma	10
	Others	15

General awareness of lazy eye was relatively balanced, with 208 (52%) participants having heard of it and 192 (48%) not. When asked if a lazy eye can be detected with the naked eye, only 94 (23.5%) correctly responded "no." A comprehensive eye examination was recognized by 166 (41.5%) as necessary for detecting lazy eye. The belief that lazy eye primarily affects children was held by 173 (43.3%). Regarding the possibility of treating lazy eye at any age, 88 (22%) correctly responded by disagreeing. Overall, 138 (34.5%) had a good level of general knowledge about amblyopia, while 262 (65.5%) exhibited a poor level of knowledge (Table 2).

**Table 2:** General awareness of lazy eye *The correct answer.

Questions	Response	N (%)
Have you ever heard of lazy eye?	Yes	208 (52)
	No	192 (48)
Can the lazy eye be detected with the naked eye?	Yes	90 (22.5)
	No*	94 (23.5)
	I don’t know	216 (54)
Lazy eye can only be detected by a comprehensive eye examination.	Yes*	166 (41.5)
	No	64 (16)
	I don’t know	170 (42.5)
Lazy eye is a disease that affects children.	Yes*	173 (43.3)
	No	42 (10.5)
	I don’t know	185 (46.2)
Lazy eyes can be treated at any age.	Yes	74 (18.5)
	No*	88 (22)
	I don’t know	238 (59.5
Knowledge level	Good	138 (34.5)
	Bad	262 (65.5)

Participants identified various causes of lazy eye, with the most commonly known cause being decreased vision in one eye, reported by 217 (54.3%). This was followed by a family history of the condition, cited by 203 (50.8%), and other eye diseases, such as hereditary lens opacity, mentioned by 191 (47.8%). Knowledge levels showed that 175 (43.8%) participants had good knowledge about lazy eye, while 225 (56.2%) had poor knowledge (Table 3).

**Table 3:** Awareness about causes of lazy eye *The correct answer.

Causes	Response	N (%)
Eye deviation (inward or outward deviation)	Yes*	161 (40.3)
	No	48 (12)
	I don’t know	191 (47.7)
Decreased vision in one eye more than in the other eye	Yes*	217 (54.3)
	No	36 (9)
	I don’t know	147 (36.7)
Eye diseases, such as hereditary lens opacity/ptosis/corneal opacity	Yes*	191 (47.8)
	No	47 (11.8)
	I don’t know	162 (40.4)
Family history of lazy eye	Yes*	203 (50.8)
	No	56 (14)
	I don’t know	141 (35.2)
Premature birth	Yes*	109 (27.3)
	No	76 (19)
	I don’t know	215 (53.7)
Use of electronic devices	Yes*	157 (39.2)
	No	75 (18.8)
	I don’t know	168 (42)
Malnutrition	Yes*	134 (33.5)
	No	77 (19.3)
	I don’t know	189 (47.2)
Knowledge level	Good	175 (43.8)
	Bad	225 (56.2)

Regarding complications, 38 (9.5%) participants incorrectly believed that lazy eye does not cause any complications. Decreased vision was recognized as a complication by 248 (62%), impaired quality of life by 228 (57%), and psychological effects by 211 (52.8%). Knowledge levels about complications showed that 159 (39.8%) participants had good knowledge, while 241 (60.2%) had poor knowledge (Table 4).

**Table 4:** Awareness about complications *The correct answer.

Complications	Response	N (%)
It does not cause any complications	Yes*	38 (9.5)
	No	182 (45.5)
	I don’t know	180 (45)
Decreased vision	Yes*	248 (62)
	No	28 (7)
	I don’t know	124 (31)
Blindness	Yes*	158 (39.5)
	No	74 (18.5)
	I don’t know	168 (42)
Loss of 3D perception	Yes*	181 (45.3)
	No	46 (11.5)
	I don’t know	173 (43.2)
Psychological	Yes*	211 (52.8)
	No	43 (10.8)
	I don’t know	146 (36.4)
Impaired quality of life	Yes*	228 (57)
	No	38 (9.5)
	I don’t know	134 (33.5)
Knowledge level	Good	159 (39.8)
	Bad	241 (60.2)

For treatment, patching was recognized by 203 (50.8%) participants, surgery by 179 (44.8%), glasses by 205 (51.3%), and eye exercises by 239 (59.8%). Overall, 195 (48.8%) participants had good knowledge about treatment, while 205 (51.2%) had poor knowledge (Table 5).

**Table 5:** Awareness about the treatment of the lazy eye *The correct answer.

Treatment	Response	N (%)
Treatment is not required	Yes	36 (9)
	No*	240 (60)
	I don’t know	124 (31)
Patching (covering the healthy eye)	Yes*	203 (50.8)
	No	50 (12.5)
	I don’t know	147 (36.7)
Surgery	Yes*	179 (44.8)
	No	68 (17)
	I don’t know	153 (38.2)
Glasses	Yes*	205 (51.3)
	No	45 (11.3)
	I don’t know	150 (37.4)
Eye exercise	Yes*	239 (59.8)
	No	38 (9.5)
	I don’t know	123 (30.7)
Knowledge level	Good	195 (48.8)
	Bad	205 (51.2)

**Figure 1:**
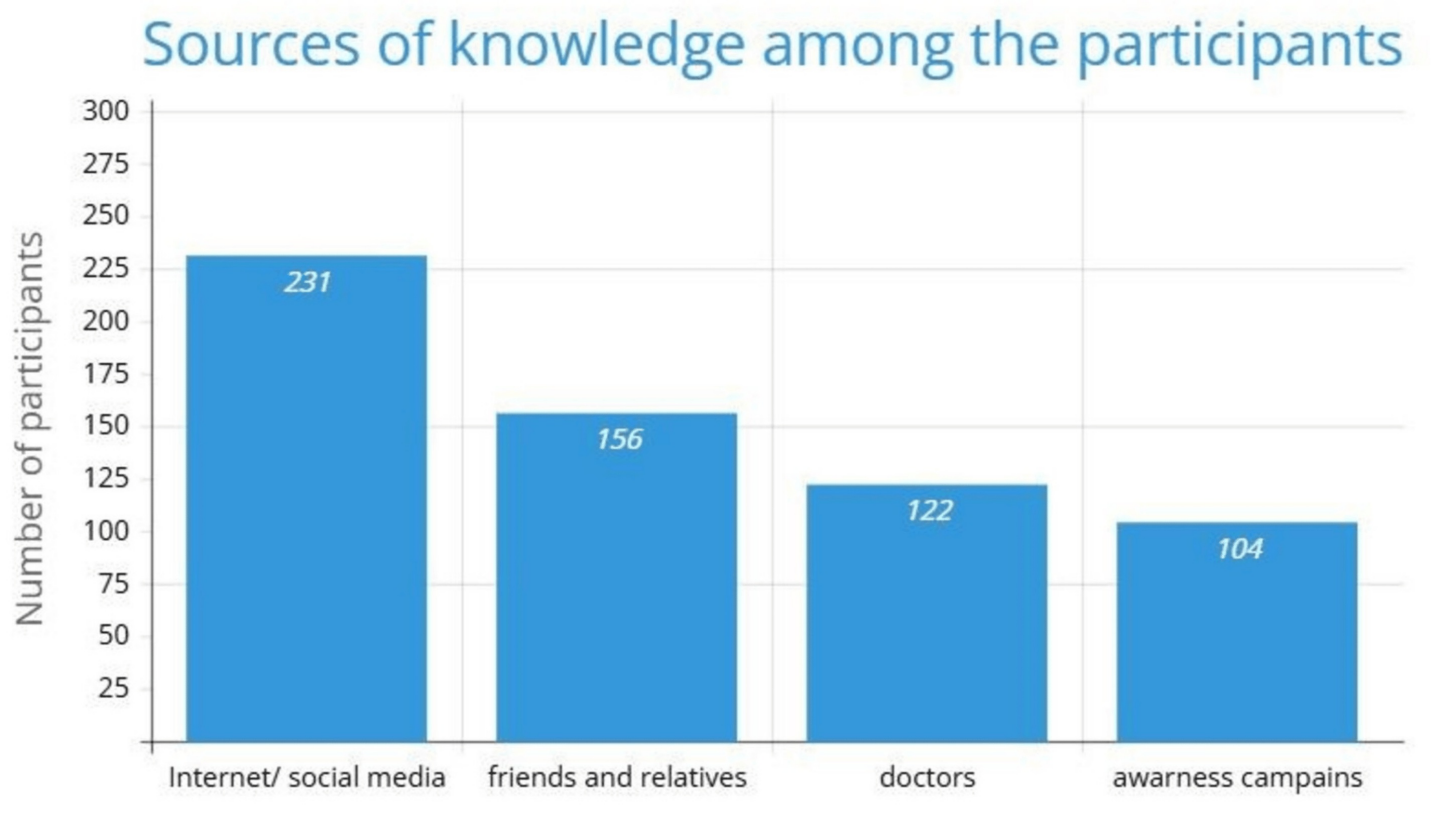
Source of knowledge

Social media and the Internet were the most common sources of information about lazy eye, cited by 231 (57.7%) participants, followed by friends and relatives, who were mentioned by 156 (39%) participants (Figure 1).

A chi-square test was used to examine the association between various sociodemographic characteristics and the level of knowledge about lazy eye among the population of the Al-Baha region. No significant associations were found, except for participants with no family history of eye diseases, who, interestingly, had a higher level of knowledge compared to those with a family history (P = 0.03) (Table 6).

**Table 6:** Association between different sociodemographic characteristics and level of knowledge of the population of the Al-Baha region

Sociodemographic variables		Knowledge level		P-value
		Good	Bad	
Gender	Male	49 (33.1)	105 (41.7)	0.09
	Female	99 (66.9)	147 (58.3)	
Age group (years)	18–29	95 (64.2)	150 (59.5)	0.2
	30–39	26 (17.6)	34 (13.5)	
	40–49	19 (12.8)	43 (17.1)	
	Above 50	8 (5.4)	25 (9.9)	
Residency	Al-Baha	58 (39.2)	87 (34.4)	0.07
	Beljurashi	25 (16.8)	26 (10.3)	
	Almakhwat	18 (12.2)	47 (18.7)	
	Al-Aqiq	18 (12.2)	47 (18.7)	
	Others	29 (19.6)	45 (17.9)	
Marital status	Single	89 (60.1)	144 (57.1)	0.8
	Married	57 (38.5)	104 (41.3)	
	Divorced	2 (1.4)	4 (1.6)	
Educational level	Primary	3 (2)	12 (4.8)	0.07
	High school	27 (18.2)	59 (23.4)	
	College bachelor	100 (67.6)	166 (65.8)	
	Master or PhD	18 (12.2)	14 (5.6)	
	Illiterate	0	1 (0.4)	
Monthly income	Less than 5000	94 (63.5)	147 (58.4)	0.5
	5000–1000	28 (18.9)	48 (19)	
	10000–15000	13 (8.8)	35 (13.9)	
	More than 15000	13 (8.8)	22 (8.7)	
Do you have children under 12 years old?	Yes	53 (35.8)	71 (28.2)	0.1
	No	95 (64.2)	181 (71.8)	
Do you have a child with lazy eyes?	Yes	12 (8.1)	10 (4)	0.08
	No	136 (91.9)	242 (96)	
Do you have a family history of eye diseases?	Yes	57 (38.5)	70 (27.8)	0.03*
	No	91 (61.5)	182 (72.2)	

## Discussion

Amblyopia is a prevalent visual impairment in children, characterized by reduced visual acuity that is not quickly corrected by refractive measures. It typically emerges in children aged seven to eight years but can be completely cured if identified and treated before nine to ten years of age. If left untreated, it can negatively impact quality of life and academic performance [11]. This study aimed to assess the knowledge and awareness of amblyopia among the adult population of Al-Baha, Saudi Arabia. The findings shed light on various aspects, including awareness levels, sources of information, understanding of etiology and treatment, and the influence of sociodemographic factors. These insights are crucial for identifying gaps in public knowledge and guiding targeted educational efforts.

Our results revealed a notably low level of awareness regarding amblyopia. Only 43.3% of participants recognized that amblyopia primarily affects children, and just 22% understood that it cannot be effectively treated beyond a certain age. Interestingly, these findings are consistent with a study conducted in Hail, Saudi Arabia, where 52.4% of participants knew the definition of amblyopia, and 43.4% were aware of its treatment [12]. This similarity underscores the widespread issue of limited awareness in the region, highlighting the need for enhanced educational campaigns across different geographical areas.

Regarding the causes of amblyopia, 43.8% of participants demonstrated a good understanding, with refractive error being the most frequently identified cause. This contrasts with other studies where genetic factors were more commonly recognized [13]. The greater familiarity with refractive error may be attributed to its common association with visual impairment, as evidenced by a previous study in Al-Baha, which identified refractive error as a major cause of visual impairment among schoolchildren [14].

While 48.8% of participants were knowledgeable about different amblyopia treatment options, a smaller proportion, 39.8%, were aware of the potential complications if left untreated. This discrepancy in knowledge aligns with the findings of a previous study conducted in Al-Baha involving parents [10]. The disparity underscores the critical need for increased awareness about the consequences of untreated amblyopia, emphasizing the importance of timely intervention.

A sociodemographic factor revealed that higher knowledge scores were associated with the female gender, single marital status, younger age, and holding a college degree. This contrasts with a study conducted in Riyadh, which found that older married women had better knowledge of amblyopia [15]. This difference may be due to the fact that the majority of our sample consisted of young, educated females.

Interestingly, participants without a family history of eye disease exhibited higher levels of knowledge compared to those with a family history. This finding suggests that individuals without genetic predispositions may engage more actively with eye health information, which could have implications for how eye disease awareness is approached in educational efforts.

It has been observed that the Internet and social media emerged as the predominant sources of information for the participants in our study. This finding aligns with the results of other studies conducted in Saudi Arabia [6, 13]. This trend offers a unique opportunity to effectively raise awareness about amblyopia by creating informative and engaging educational videos and disseminating them through trusted and widely used online platforms.

Limitations: Despite these strengths, the study has notable limitations. Its cross-sectional design restricts causal inference and does not capture changes in awareness over time or the impact of potential interventions. The focus on the Al-Baha region may limit the generalizability of the findings to other regions or populations, as different areas may exhibit varying levels of awareness and information sources. Additionally, the predominance of female participants introduces a potential selection bias, which could affect the representativeness of the results. The sample’s limited demographic diversity in terms of age, marital status, and educational background also restricts the breadth of the findings. Future research should address these limitations by employing longitudinal designs to track changes in awareness, including more diverse and representative samples, and exploring the effects of targeted educational interventions on different demographic groups.

## Conclusions

Our study assessed the knowledge and awareness of amblyopia among the population of Al-Baha, revealing a higher percentage of individuals with low levels of knowledge. This finding underscores the need for additional education and targeted interventions, such as awareness campaigns and workshops. Future research should focus on implementing educational interventions and assessing knowledge levels, with a particular emphasis on allocating more resources to improve awareness in the Al-Baha region.
